# Supplementary material for: Transport of metformin metabolites by guanidinium exporters of the small multidrug resistance family
Source: J Gen Physiol. 2024 Jan 31;156(3):e202313464. doi: 10.1085/jgp.202313464 (PMC10829512; doi:10.1085/jgp.202313464)
Supplement: Table S1 — shows coding sequences for transporters examined in this study. [file JGP_202313464_TableS1.docx]

**Supplementary Table 1. Coding sequences for transporters examined in this study.**

| **Construct** | **Coding Sequence** |
| --- | --- |
| Gdx-Clo | **ATGGCGTGGCTGATCCTGATCATTGCGGGTATTTTCGAGGTGGTTTGGGCGATCGCGCTGAAGTACAGCAACGGTTTTACCCGTCTGATCCCGAGCATGATCACCCTGATTGGCATGCTGATTAGCTTCTACCTGCTGAGCCAAGCGACCAAGACCCTGCCGATTGGTACCGCGTATGCGATCTGGACCGGTATTGGCGCGCTGGGTGCGGTGATTTGCGGCATCATTTTCTTTAAAGAACCGCTGACCGCGCTGCGTATCGTTTTTATGATTCTGCTGCTGACCGGTATCATTGGCCTGAAAGCGACCAGCAGC**GGTGGCACCGCGAAAGCGAGCCTGGTGCCGCGTGGTAGCGGTGGCCACCACCACCACCACCACTG |
| Gdx-Eco | **ATGAGCTGGATCATTCTGGTGATCGCGGGCCTGCTGGAAGTGGTTTGGGCGGTTGGCCTGAAGTACACCCACGGTTTCAGCCGTCTGACCCCGAGCGTGATTACCGTTACCGCGATGGTGGTTAGCATGGCGCTGCTGGCGTGGGCGATGAAAAGCCTGCCGGTGGGTACCGCGTATGCGGTTTGGACCGGTATTGGTGCGGTGGGTGCGGCGATCACCGGCATTGTTCTGTTTGGTGAAAGCGCGAACCCGATGCGTCTGGCGAGCCTGGCGCTGATTGTGCTGGGTATCATTGGCCTGAAGCTGAGCACCCAT**GGTGGCACCGCGAAAGCGAGCCTGGTTCCGCGTGGCAGCGGTGGCCACCACCACCACCACCACTGA |
| Gdx-pPro | ATGGGCAGCAGCCATCACCATCATCATCACAGCAGCGGCCTGGTGCCGCGCGGCAGCGGTGGCACCGCGAAAGCGAGC**TCCTGGATCGTTCTGTTGATCGCGGGCTTGTTAGAGGTTGTATGGGCCATCGGACTTAAATACACTCACGGTTTCACACGCTTGACCCCAAGTATTATTACAATCGCTGCTATGATCGTGTCAATCGCCATGCTTTCGTGGGCAATGCGCACCCTTCCAGTCGGGACGGCATACGCAGTGTGGACCGGGATTGGCGCTGTTGGAGCGGCGATCACCGGCATCCTTCTTCTGGGTGAAAGTGCGTCACCGGCTCGTTTGTTGAGTCTTGGACTGATTGTGGCCGGGATTATCGGCTTAAAACTTAGCACTCATTAA** |
| Gdx-pAmi | ATGGGCAGCAGCCATCACCATCATCATCACAGCAGCGGCCTGGTGCCGCGCGGCAGCGGTGGCACCGCGAAAGCGAGC**GCTTGGATCTATTTACTGCTTGCTGGTCTTTTCGAGATTGGTTGGCCGGTCGGACTGAAGATGGCTCAAGAGCCTGACACGCGTTGGAGTGGGATTGGTGTCGCAGTGGTATTTATGGGAATTAGCGGGGCGTTGCTTTTCCTTGCTCAGCGCACCATTCCCATTGGCACTGCTTACGCTATCTGGACCGGAATTGGAGCCGCCGGAACCTTTTTAGTTGGAGTTATGTACTACGGCGACCCCACTAGCTTCTTTCGTTATTTGGGTGTCGCACTTATCGTTGCTGGAGTGGCGACACTGAAGTTAGCGCACTAA** |
| EmrE | ATGGGCAGCAGCCATCACCATCATCATCACAGCAGCGGCCTGGTGCCGCGCGGCAGCCAT**ATGAACCCTTATATTTATCTTGGTGGTGCAATACTTGCAGAGGTCATTGGTACAACCTTAATGAAGTTTTCAGAAGGTTTTACACGGTTATGGCCATCTGTTGGTACAATTATTTGTTATTGTGCATCATTCTGGTTATTAGCTCAGACGCTGGCTTATATTCCTACAGGGATTGCTTATGCTATCTGGTCAGGAGTCGGTATTGTCCTGATTAGCTTACTGTCATGGGGATTTTTCGGCCAACGGCTGGACCTGCCAGCCATTATAGGCATGATGTTGATTTGTGCCGGTGTGTTGATTATTAATTTATTGTCACGAAGCACACCACATTAA** |
| QacE | ATGGGCAGCAGCCATCACCATCATCATCACAGCAGCGGCCTGGTGCCGCGCGGCAGC**AAAGGTTGGCTTTTTCTTGTAATTGCTATCGTAGGTGAAGTAATCGCTACCTCGGCCTTAAAATCAAGCGAAGGTTTTACCAAACTTGCGCCCTCGGCTGTCGTCATTATCGGATACGGAATTGCGTTCTATTTCCTGTCATTAGTGATGAAGTCTATTCCGGTGGGGGTTGCTTATGCGGTATGGAGTGGGCTTGGAGTTGTAATTATTACCGCAATCGCTTGGCTTCTTCACGGTCAGAAGTTGGACGCCTGGGGCTTCGTAGGTATGGGCCTTATTGTCAGTGGAGTCGTCGTTTTAAATTTGCTGTCCAAAGCGTCGGCACATTAA** |

**Gene sequence**

His_6_-Tag

Cleavage Site
