# Supplementary material for: Transport of metformin metabolites by guanidinium exporters of the small multidrug resistance family
Source: J Gen Physiol. 2024 Jan 31;156(3):e202313464. doi: 10.1085/jgp.202313464 (PMC10829512; doi:10.1085/jgp.202313464)
Supplement: Table S2 — shows reconstitution efficiencies of SMRGdx homologs assessed by quantitative Western blot. [file JGP_202313464_TableS2.docx]

**Supplementary Table 2. Reconstitution efficiencies of SMR_Gdx_ homologues assessed by quantitative Western blot.** The value F_Gdx_/F_Fluc_ is an arbitrary number comparing florescence intensities in bands of co-reconstituted samples.

|  | F_Gdx_/F_Fluc_ | p (vs Gdx-Clo) | p (vs Gdx-Eco) | p (vs Gdx-pPro) | p (vs Gdx-pAmi) |
| --- | --- | --- | --- | --- | --- |
| Gdx-Clo | 4.54±0.69 |  | 0.15 (ns) | 0.17 (ns) | .017 |
| Gdx-Eco | 6.65±1.28 | 0.15 (ns) |  | 0.67 (ns) | .03 |
| Gdx-pPro | 7.59±2.17 | 0.17 (ns) | 0.67 (ns) |  | .01 |
| Gdx-pAmi | 0.44±0.13 | .017 | .03 | .01 |  |
