# Supplementary material for: Transport of metformin metabolites by guanidinium exporters of the small multidrug resistance family
Source: J Gen Physiol. 2024 Jan 31;156(3):e202313464. doi: 10.1085/jgp.202313464 (PMC10829512; doi:10.1085/jgp.202313464)
Supplement: SourceData F4 — is the source file for Fig. 4. [file JGP_202313464_SourceDataF4.pdf]

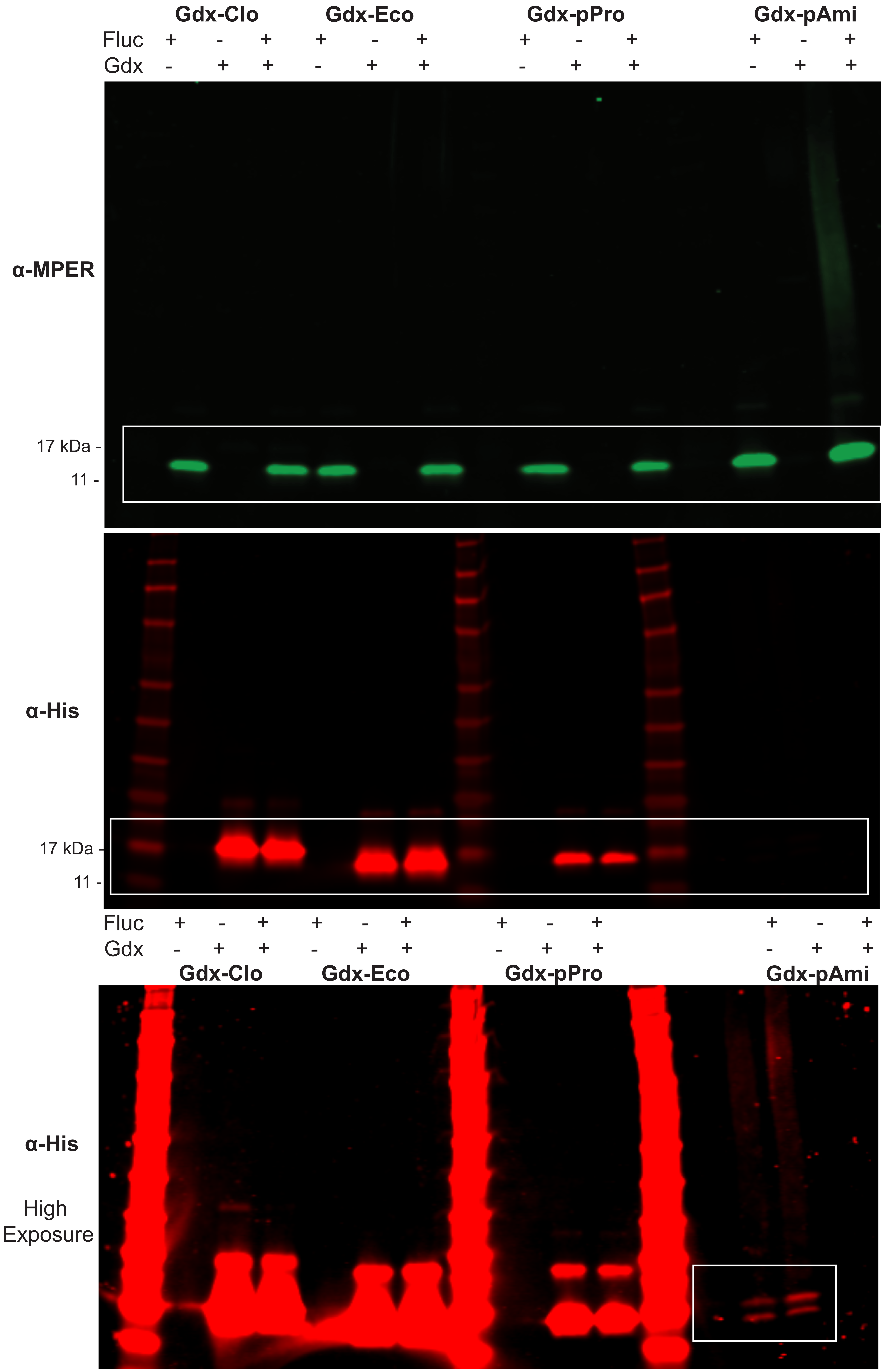

Gdx-Clo
Gdx-Eco
Gdx-pPro
Gdx-pAmi
Fluc + - + + - + + - + + - +
Gdx - + + - + + - + + - + +
α-MPER
17 kDa -
11 -
α-His
17 kDa -
11 -
Fluc + - + + - + + - + + - +
Gdx - + + - + + - + + - + +
Gdx-Clo
Gdx-Eco
Gdx-pPro
Gdx-pAmi
α-His
High Exposure
